# Optimizing Mechanical and Thermal Properties of Slag-Based Geopolymer Fiber Boards via Fiber Pretreatment and Reinforcement Type

**DOI:** 10.3390/polym18030423

**Published:** 2026-02-06

**Authors:** Sebnem Sevil Arpaci, Ergun Guntekin

**Affiliations:** Department of Forest Industry Engineering, Bursa Technical University, Bursa 16310, Turkey

**Keywords:** geopolymer, ground granulated blast furnace slag, fiber board, mechanical and physical properties, sustainable building materials

## Abstract

This study aims to optimize the physical, mechanical, and thermal properties of 100% Ground Granulated Blast Furnace Slag (GGBFS) based geopolymer wood-composite panels. Pine fibers were utilized as the primary reinforcement matrix, while glass and hemp fibers were introduced as secondary reinforcements at varying proportions (3%, 6%, 9% by weight). The research investigated the effects of fiber pretreatments (hot water vs. 1% NaOH) and reinforcement hybridization. Results indicate that GGBFS successfully geopolymerized, forming a hybrid N-A-S-H and C-A-S-H gel network. Quantitative analysis revealed that 9% glass fiber reinforcement yielded the highest mechanical performance, achieving a Modulus of Rupture (MOR) of 10.05 N/mm^2^ and Internal Bond (IB) strength of 1.32 N/mm^2^, alongside superior water resistance (1.0% Thickness Swelling). Conversely, while hemp fiber inclusion reduced mechanical strength (MOR: 5.77 N/mm^2^ at 9%), it significantly enhanced thermal insulation, reducing thermal conductivity to 0.10 W/m·K. It was observed that aggressive NaOH pretreatment caused alkali-induced degradation of pine fibers, negatively impacting the composite’s integrity compared to hot water treatment. This study demonstrates the feasibility of tailoring 100% slag-based geopolymer composites for either structural (glass-reinforced) or insulating (hemp-reinforced) applications using industrial by-products.

## 1. Introduction

Global climate change, standing as one of the most pressing threats to our planet, serves as an unavoidable wake-up call, compelling every industry to transition toward sustainable practices [1]. At the heart of this challenge lies the construction sector, heavily scrutinized for its massive energy consumption and environmental footprint [2]. Research indicates that this sector is responsible for approximately 35% of global carbon dioxide (CO_2_) emissions [1]. The primary culprit is Portland cement, the production of which accounts for roughly 8% of global emissions [3]. These statistics expose the unsustainable nature of traditional building materials and underscore the critical need for a fundamental shift in construction methodologies.

This urgency has driven researchers to seek viable alternatives to cement. Initially, efforts focused on reducing cement consumption by incorporating industrial by-products, such as fly ash and GGBFS, into mixtures [4]. However, a true paradigm shift occurred with the development of entirely cement-free binders [5]. Geopolymers, introduced by Joseph Davidovits in the 1970s [6], have emerged as a ground-breaking substitute. Driven by the demand for energy-efficient materials, recent research has focused on transforming geopolymer matrices into high-performance composites through reinforcement with waste-derived or natural fibers [7,8].

Nevertheless, developing fiber-reinforced geopolymer composites—especially utilizing waste fibers—presents challenges. Unlike fly ash-based systems, slag-based geopolymers are prone to high drying shrinkage, which can lead to micro-cracking and debonding at the fiber-matrix interface. Furthermore, the mismatch between the hydrophilic nature of natural fibers and the highly alkaline environment (pH > 13) of the geopolymer matrix often results in weak interfacial bonding [9]. Recent literature emphasizes that while fiber treatment can improve mechanical properties and minimize water absorption, excessive chemical exposure may degrade the fiber structure [9]. Therefore, optimizing pretreatment is crucial to balance surface cleaning with fiber integrity.

To address this, studies have explored pretreatments such as alkali processing or hot water immersion. Previous investigations have explored wood fiber reinforcements in various matrices [10]. However, a significant gap remains regarding the “optimization of pretreatment severity” specifically for 100% slag-based systems, which exhibit more aggressive reaction kinetics than fly ash blends. This study addresses this gap by engineering a fully waste-derived, sustainable geopolymer panel. The main novelty lies in identifying the threshold where chemical pretreatment shifts from beneficial modification to detrimental degradation in a high calcium geopolymer environment.

## 2. Materials and Methods

### 2.1. Binder and Chemical Activators

GGBFS, sourced from an iron and steel plant in the Samsun region, served as the primary binder. Its chemical composition was characterized using an X-ray fluorescence (XRF) spectrometer (RIGAKU-SUPERMINI 200, Rigaku Corporation, Tokyo, Japan). Consistent with high-reactivity precursors described in the literature, the slag exhibited a Blaine fineness of approximately 4250 cm^2^/g.

For alkaline activation, a combination of sodium silicate (Na_2_SiO_3_) and sodium hydroxide (NaOH) was employed. The commercial Na_2_SiO_3_ solution (Merck, Darmstadt, Germany) contained 26.5% SiO_2_ and 8% Na_2_O by weight (pH 11.36, density 1.35 g/cm^3^), yielding a silica modulus (Ms) of 3.42. NaOH pellets (Merck) were dissolved in distilled water to prepare an 8 Molar (8 M) solution, which was allowed to cool to room temperature prior to use.

The activator solution was prepared by mixing the Na_2_SiO_3_ and 8 M NaOH at a weight ratio of 2.5:1. This mixture was stirred for 5 min and left to rest for 24 h to ensure thermal equilibrium and complete depolymerization of silica species. The resulting solution possessed an effective Ms of approximately 1.55—falling within the optimal range (1.0–2.0) for high-strength geopolymers [11].

### 2.2. Reinforcement Fibers and Characterization

**Figure 1:**
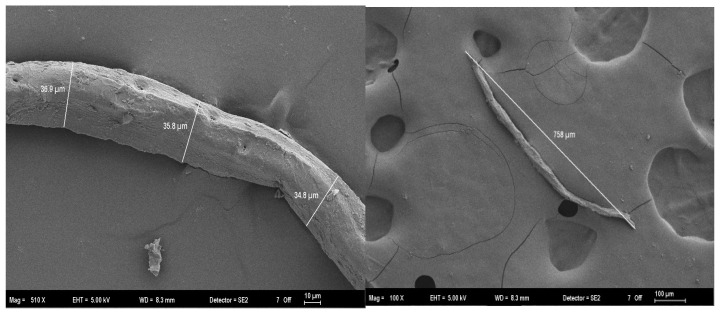
SEM micrographs showing the morphology and dimensions of the softwood (pine) fibers.

Softwood (pine) fibers (average length 0.5–1 mm; diameter ~34–37 µm), obtained from the Kastamonu Entegre Gebze Facility (Istanbul, Turkey), were selected as the primary reinforcement elements (Figure 1).

To create a hybrid composite structure, hemp and glass fibers were introduced as additives at 3%, 6%, and 9% by weight of the primary binder.

**Figure 2:**
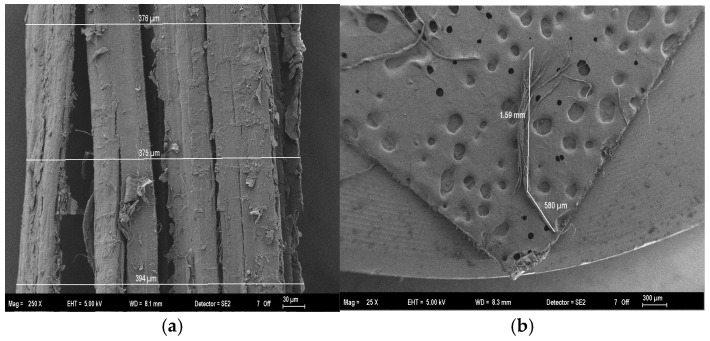
Morphological characterization of Hemp Fibers (HF): (**a**) SEM micrograph showing the width and surface texture of a fiber bundle; (**b**) Macro-optical view of the raw hemp fibers and the ruler used for length reference.

Hemp fibers (HF), utilized as organic additives, are shown in their raw form in Figure 2b. The fibers were manually cut to a length of approximately 1.6 cm prior to incorporation into the matrix. SEM analysis (Figure 2a) revealed the microstructure of the fiber bundles, which exhibited widths ranging from approximately 376 to 394 μm, while the individual technical fibers possessed an average diameter of 94 μm. Their chemical constituents were likewise determined experimentally. Commercial E-glass fibers (GF) (length 12 mm, diameter 13–15 μm) served as inorganic reference additives, with technical properties adopted as reported by the supplier [12].

The pine fibers constituted the main reinforcement network. Their chemical composition (cellulose, hemicellulose, and lignin), along with that of the hemp fibers, was experimentally analyzed according to standard wet chemistry protocols [13] to assess suitability.

### 2.3. Fiber Surface Pretreatments

To mitigate the adverse effects of amorphous constituents—such as hemicellulose, pectin, and waxes—on fiber-matrix adhesion, surface pretreatments were applied specifically to the pine wood fibers. Conversely, the hemp and glass fibers were incorporated in their as-received state.

Two distinct pretreatment protocols were evaluated, both maintaining a solid-to-liquid ratio of 1:9 to ensure dispersion.

- Hydrothermal Treatment: Fibers were immersed in distilled water at 80 °C for 24 h to solubilize starch and water-soluble extractives.
- Alkaline Treatment: Fibers were subjected to a 1% NaOH solution at room temperature for 24 h (mercerization). Hemicellulose is highly sensitive to alkaline environments; its removal is intended to increase the cellulose content and roughness of the surface, but excessive removal can weaken the fiber structure.

Following chemical exposure, the fibers were washed with distilled water until a neutral pH (7.0 ± 0.5) was achieved. Excess water was removed via filtration, and fibers were conditioned to an equilibrium moisture content of 10–12% (20 ± 2 °C, 65 ± 5% RH).

### 2.4. Preparation of Composite Panels

#### 2.4.1. Mix Design and Proportions

The composite panels were designed with a target density of 1.3 g/cm^3^. A total of nine different board types were manufactured based on fiber pretreatment and additive variations. The specific experimental design, including the control group (G) and the modified series (G1–G8), is detailed in Table 1.

The composite panels were designed with a target density of 1.3 g/cm^3^ and final dimensions of 350 × 300 × 12 mm. A fixed activator-to-binder ratio of 0.33 (1:3 by weight) was adopted for all mixtures to ensure standardization. However, the effective liquid content was inherently influenced by the residual moisture in the pretreated fibers and the external application of the 100 mL calcium chloride (CaCl_2_) accelerator spray. These variables were carefully managed during fabrication to guarantee consistent workability and mat consolidation. Based on optimal parameters from preliminary trials, the primary reinforcement (pine fibers) was incorporated at a fiber-to-binder weight ratio of 1:9. For hybrid formulations, the additive fiber content was calculated relative to the weight of the GGBFS binder to ensure consistency across all batches.

#### 2.4.2. Fabrication Process

The fabrication began by mixing the weighed GGBFS and alkaline activator solution in a mechanical mixer for 5 min to achieve a homogeneous paste.

Prior to their addition to the matrix, a specific interfacial modification was applied to the fibers. The wood fibers (and additive fibers, where applicable) were moistened by spraying with a CaCl_2_ solution. The dosage was set at 5% CaCl_2_ by weight of the GGBFS. This step aimed to strengthen the fiber-matrix bond by promoting the formation of calcium-based deposits at the interface [14]. The total volume of the spray solution was fixed at 100 mL for all batches; this volume, along with the initial moisture content of the wood fibers (8.5%), was factored into the overall water-to-binder ratio calculation to maintain rheological stability.

Immediately following the spray treatment, the fibers were gradually added to the binder paste. Mixing continued for an additional 5 min until the fibers were uniformly dispersed within the matrix.

#### 2.4.3. Molding and Curing

The fresh geopolymer-fiber mixture was manually spread into stainless steel molds to ensure uniform distribution. The filled molds were then subjected to a pressure of 3 MPa using a cold press at room temperature (23 ± 2 °C) for a duration of 24 h. This specific pressure was determined through preliminary optimization to achieve maximum compaction without compromising the cellular integrity of the wood fibers.

Following the pressing stage, the panels were demolded and transferred to an oven for final curing. The samples were cured at 40 °C for 6 h; a regime selected to facilitate strength development while preventing the micro-cracking and shrinkage often induced by higher temperatures in slag-dominated systems. After curing, the panels were stored at ambient conditions until testing.

### 2.5. Microstructural and Chemical Characterization Methods

#### 2.5.1. Physical Property Tests

- **Density (d):** The density of the composite panels was determined in accordance with the TS EN 323 [15] standard. The mass of each specimen was measured using an analytical balance (±0.001 g precision), while volumes were calculated from dimensions taken with a digital caliper (±0.01 mm). The reported density represents the average of three specimens per panel group.
- **Water Absorption (WA) and Thickness Swelling (TS):** Dimensional stability was evaluated based on the TS EN 317 [16] standard following 24 h of water immersion. Prior to testing, eight replicate samples were conditioned at 20 ± 2 °C and 65 ± 5% relative humidity. Changes in mass and thickness were recorded to calculate WA and TS, respectively. Results are presented as the arithmetic mean of the eight replicates.
- **Thermal Conductivity:** Thermal conductivity coefficients were measured using a FOX 314 Heat Flow Meter in accordance with ASTM C-518 [17]. A constant temperature gradient was established by setting the cold and hot plates to 10 °C and 30 °C, respectively. Specimens (100 × 100 × 10 mm) were tested within an insulated guard area to minimize edge heat losses.

#### 2.5.2. Mechanical Testing Procedures

- **Modulus of Rupture (MOR) and Modulus of Elasticity (MOE):** Three-point bending tests were performed according to TS EN 310 [18]. The support span was set to 20 times the specimen thickness. A loading rate of approximately 4 mm/min was applied to ensure failure occurred within 60 ± 30 s. The MOR was calculated based on the maximum load recorded at failure, while the MOE was determined from the slope of the linear elastic region of the load-deflection curve. Eight specimens were tested for each group.
- **Internal Bond (IB) Strength:** Tensile strength perpendicular to the board plane was assessed using 50 × 50 mm specimens in accordance with TS EN 319 [18]. The tensile load was applied at a rate of 0.6 mm/min until failure. The maximum force was recorded, and the average of eight replicates was reported

#### 2.5.3. Microstructural and Chemical Analyses

**FTIR:** To identify functional groups and chemical bonding, FTIR analysis was conducted on powdered samples obtained from fracture surfaces. Prior to analysis, samples were dried at 80 °C for 24 h. Spectra were acquired using a Bruker Tensor 37 spectrometer (Bruker Optik GmbH, Ettlingen, Germany) equipped with an ATR module, scanning from 4000 to 400 cm^−1^ at a resolution of 4 cm^−1^ (32 scans).

**XRD:** The crystallographic structure of the cured geopolymer binder was investigated using a Bruker D8 Advance diffractometer (Bruker, Karlsruhe, Germany) (Cu-Kα radiation). Fragments from fractured specimens were vacuum-dried at 80 °C for 24 h prior to analysis. Scans were performed over a 2θ range of 10–100° at a rate of 0.5°/min.

**SEM:** The morphology of the fiber-matrix interface and composite microstructure was examined using a Carl Zeiss Gemini 300 SEM (Carl Zeiss, Oberkochen, Germany). Specimens (~1 mm^3^) were vacuum-dried at 60 °C for 24 h and sputter-coated with a gold-palladium layer (Leica ACE600, Leica Microsystems, Vienna, Austria; 20 mA, 30 s) to prevent charging.

#### 2.5.4. Statistical Analysis

Data were statistically analyzed using IBM SPSS Statistics 22 software. A one-way analysis of variance (ANOVA) was employed to assess the significance of differences between groups. For factors exhibiting statistical significance (*p* < 0.05), Tukey’s multiple comparison test was applied to identify specific differences among group means

## 3. Results and Discussion

### 3.1. Chemical Characterization of Raw Materials

The chemical composition of the GGBFS, determined via XRF analysis, is presented in Table 1. The slag is predominantly composed of calcium oxide (CaO), silica (SiO_2_), and alumina (Al_2_O_3_), which collectively constitute the backbone of the geopolymer network (Table 2).

The final performance of geopolymer systems is intrinsically linked to the mineralogical makeup of the precursor. As shown in Table 1, the GGBFS used in this study possesses a naturally high Si/Al ratio. Theoretically, this favors the formation of a highly cross-linked N-A-S-H network. However, the substantial CaO content (38.5%) introduces a secondary reaction pathway upon alkaline activation, leading to a hybrid gel system where calcium-rich C-A-S-H gels coexist with N-A-S-H gels. This hybrid structure is critical; as noted by Garcia et al. [19], C-A-S-H gel typically comprises 70–75% of the binder volume in silicate-activated slags, providing rapid setting and high early-age strength, while the N-A-S-H network contributes to workability and long-term durability [20].

In addition to the binder, the chemical constituents of the lignocellulosic reinforcements (Pine and Hemp fibers) play a decisive role in composite durability, particularly regarding their interaction with the alkaline matrix. The chemical composition of the fibers used in this study was experimentally determined and is summarized in Table 3.

As seen in Table 3, the pine fibers exhibit a typical softwood composition with a balanced lignin content (27.9%), which contributes to the rigidity of the fiber cell wall. However, the significant hemicellulose fraction (25.4%) necessitates the surface pretreatments applied in this study to prevent interfacial interference during geopolymerization.

### 3.2. Physical Properties

The physical characteristics of the produced geopolymer panels, including d, TS, WA, and thermal conductivity are summarized in Table 4. The results reveal distinct trends driven by the type of fiber reinforcement and the applied pretreatments.

#### 3.2.1. Density

**Figure 3:**
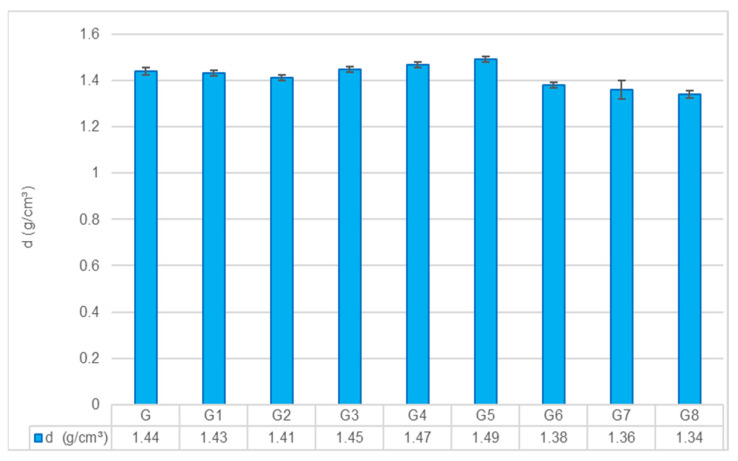
Variation in density values across different geopolymer composite groups (Error bars represent standard deviation).

The potential of geopolymer composites as lightweight construction materials is strongly dependent on the final density, as this property directly influences both structural efficiency and handling performance. The mean density values for all groups are presented in Figure 3.

A one-way ANOVA confirmed that the differences among groups were statistically significant (*p* < 0.001), indicating that both fiber type and pretreatment methods exert pronounced effects on the composite density. Tukey’s HSD post hoc test further revealed distinct homogeneous subsets; notably, the highest-density group (G5) differed significantly from the lowest-density group (G8) (*p* < 0.001).

The G exhibited a reference density of 1.44 g/cm^3^. Although the initial mix design targeted a density of 1.30 g/cm^3^, the experimental values ranged between 1.34 and 1.49 g/cm^3^. This deviation is attributed to variations in fiber dispersion within the matrix. Specifically, in natural fiber-reinforced groups, the inherent tendency of fibers to agglomerate can create micro-voids that prevent full compaction, while in glass fiber groups, the higher specific gravity of the reinforcement naturally elevates the bulk density. Similar deviations (~10%) have been reported in the literature [8,21], where rheological changes induced by high fiber loading were found to hinder the achievement of theoretical density targets.

**Effect of Pretreatments:** The G1 showed a density of 1.43 g/cm^3^, which was statistically indistinguishable from the Control (*p* = 0.999). This suggests that hot water treatment acts as a mild intervention, removing surface impurities without altering the fiber’s structural integrity or the matrix compactness. In contrast, G2 exhibited a reduced density of 1.41 g/cm^3^. Although the difference from the Control was not statistically significant (*p* = 0.332), G2 was significantly less dense than the glass fiber groups G4 and G5. This trend aligns with the “alkali-induced degradation” hypothesis. The combination of the NaOH pretreatment and the highly alkaline geopolymer matrix appears to exceed the threshold for beneficial modification, triggering the dissolution of hemicellulose and the partial hydrolysis of cellulose chains. Unlike mineralization, which would increase density, this degradation creates interfacial micro-voids, reducing the overall density.

**Effect of Fiber Reinforcement:** GF reinforcement resulted in a monotonic increase in density, consistent with the rule of mixtures. The density rose from 1.44 g/cm^3^ (G3) to 1.49 g/cm^3^ (G5) as the fiber content increased to 9%. The G5 group was significantly denser than the Control (*p* = 0.003) and all natural fiber groups. This is directly attributed to the higher specific gravity of glass fibers compared to the geopolymer matrix and their ability to disperse without absorbing water from the mix.

Conversely, HF reinforcement led to a systematic reduction in density: 1.38 g/cm^3^ (G6), 1.36 g/cm^3^ (G7), and 1.34 g/cm^3^ (G8). All HF groups were significantly lighter than the Control (*p* < 0.001). This “lightweighting” effect is primarily due to two factors: the naturally porous lumen structure of hemp fibers and the phenomenon of fiber agglomeration. As fiber content increased, the difficulty in achieving a homogeneous mix likely led to air entrapment within fiber bundles, fostering a more porous microstructure [22]. While this reduction in density compromises mechanical load-bearing capacity, it offers a distinct advantage for applications requiring lighter building panels.

#### 3.2.2. Dimensional Stability (Water Absorption and Thickness Swelling)

**Figure 4:**
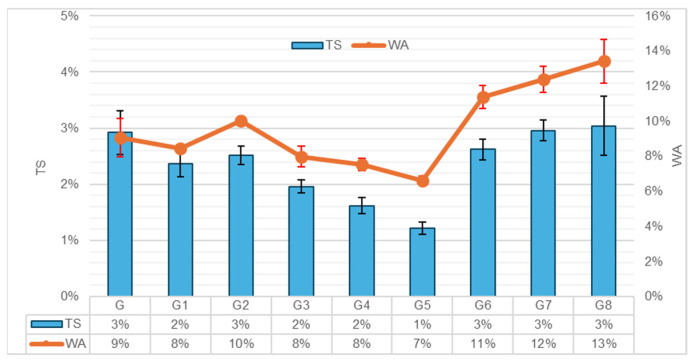
WA and TS percentages after 24 h (Error bars represent standard deviation).

The interaction between the composite material and moisture is a defining factor for its long-term durability. The 24 h WA and TS tests were conducted to evaluate the internal pore structure and the quality of the fiber–matrix interface. The results are summarized in Figure 4.

The G exhibited a TS of 3.0% and WA of 9.0%, serving as the baseline. The results indicate that dimensional stability is heavily influenced by the type of reinforcement, while the effect of pretreatments remained statistically limited under the tested conditions.

**Effect of Pretreatments:** The G1 group showed a slight improvement with 2.0% TS and 8.0% WA. However, Tukey’s post hoc analysis revealed that these differences were not statistically significant compared to the control (*p* = 0.300 for TS; *p* = 0.905 for WA). This suggests that while hot water effectively cleans surface dust, it does not sufficiently alter the hydrophilicity of the fibers to cause a measurable shift in macro-scale dimensional stability.

Conversely, the G2 group yielded values of 3.0% TS and 10.0% WA. Although these values were statistically similar to the control (*p* > 0.05), the slight upward trend in water absorption corroborates the “cumulative alkali degradation” hypothesis proposed in the density analysis [21]. The micro-structural damage and increased porosity—previously identified as the primary cause for density loss—appear to enhance the capillary network here, thereby facilitating greater water ingress.

**Effect of Fiber Reinforcement:** In contrast, HF reinforcement resulted in a marked increase in water absorption. The WA values rose linearly with fiber content, reaching 13.0% in the G8 group (*p* < 0.001 compared to G). This behavior is driven by the intrinsic hydrophilicity of natural fibers and their hollow lumen structure, which acts as a reservoir for water storage [23]. This behavior is consistent with the findings of [9], which suggest that while treatment helps, the inherent porosity of natural fibers remains a challenge in wet environments. The observed reduction in density and increased water absorption in the wood-based matrix are consistent with the findings of Duan et al., 2016 [24]. They attributed this behavior to the inherent porosity and hydrophilic nature of the cellulosic structure, which tends to retain moisture within the lumen channels despite the dense geopolymer network. Furthermore, the fiber agglomeration observed in the density analysis likely introduced macro-voids, creating an interconnected pore network that accelerates water uptake.

#### 3.2.3. Thermal Conductivity (λ)

**Figure 5:**
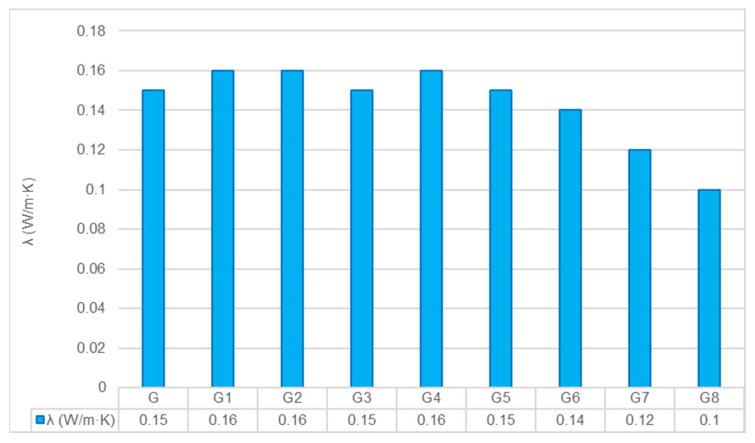
Comparison of thermal conductivity values across different composite groups.

Energy efficiency in buildings is directly linked to the thermal insulation capacity of the construction materials used. In this study, the thermal conductivity of the geopolymer composites was evaluated to determine their potential as insulating panels. The results, presented in Figure 5, highlight a clear divergence in performance driven by the reinforcement type.

**Effect of Fiber Reinforcement:** The most remarkable finding is the systematic improvement in insulation properties with the addition of HF. The thermal conductivity decreased linearly as the HF content increased, reaching a minimum of 0.10 W/m·K in the G8 group. This represents a substantial 33% reduction compared to the control matrix (0.15 W/m·K).

This enhancement is directly linked to the density reduction and porosity increase discussed in Section 3.2.1. Lignocellulosic hemp fibers possess a hierarchical structure containing a hollow lumen filled with stagnant air. Since air has an extremely low thermal conductivity (~0.026 W/m·K), these fibers effectively introduce millions of insulating micro-pockets into the matrix. Furthermore, the macro-voids created by fiber agglomeration act as additional barriers to heat flux. This mechanism—where the introduction of porous bio-fillers disrupts the conductive path of the geopolymer matrix—is well-supported by recent studies on agricultural waste-based geopolymers [21,25].

In contrast, the inclusion of GF resulted in values ranging between 0.15 and 0.16 W/m·K, showing no improvement over the control. Unlike hemp, glass fibers are solid, dense, and inorganic materials with higher intrinsic thermal conductivity. Consequently, they facilitate heat transfer via solid-state conduction rather than impeding it. This stark contrast demonstrates that for applications where thermal insulation is the primary objective, porous natural fibers are structurally superior to dense synthetic reinforcements.

**Effect of Pretreatments:** An interesting observation is that both the G1 and G2 treated groups exhibited a slight increase in thermal conductivity (0.16 W/m·K) compared to the control (0.15 W/m·K). While this might initially seem contradictory to the density loss observed in the G2 group, it can be explained by the modification of the fiber–matrix interface.

Pretreatments remove surface impurities such as waxes and dust, allowing the geopolymer paste to wet the fiber surface more effectively. This improved physical contact reduces the “interfacial thermal resistance” (also known as Kapitza resistance), creating a more continuous bridge for heat flow (phonons) between the matrix and the fiber [26]. Additionally, in the case of the G2 group, the slight increase in thermal conductivity can be attributed to the modification of the fiber–matrix interface. The alkali treatment effectively removed surface impurities such as waxes and pectins, thereby improving the wettability of the fibers. This enhanced physical contact likely reduced the interfacial thermal resistance (Kapitza resistance) between the reinforcement and the geopolymer matrix, facilitating more efficient heat transfer, despite the observed reduction in bulk density.

#### 3.2.4. Mechanical Properties (MOE, MOR, and IB)

The mechanical performance of the composites, characterized by MOE, MOR, and IB strength, is presented in Table 5. These parameters are critical for determining the structural suitability of the panels.

The results reveal a distinct dichotomy between the reinforcement types. While glass fibers reinforced the matrix, hemp fibers led to a reduction in mechanical strength, highlighting a clear trade-off between thermal insulation and load-bearing capacity.

#### 3.2.5. Flexural Performance (MOR and MOE)

**Figure 6:**
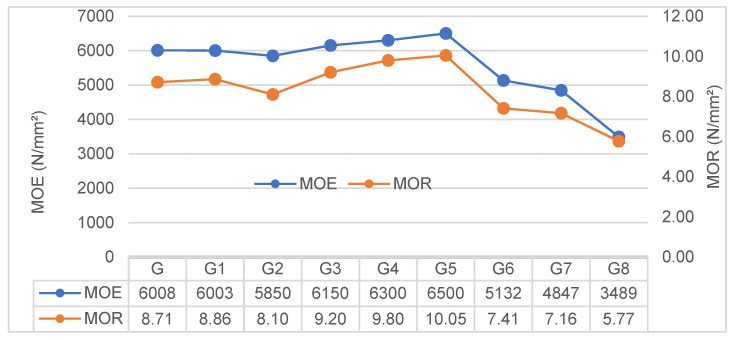
Comparison of Modulus of Elasticity (MOE) and Modulus of Rupture (MOR) trends across composite groups.

The flexural behavior of the composites, characterized by MOR and MOE, is presented in Figure 6. Since both parameters are intrinsically linked to the fiber-matrix interaction and microstructural integrity, they exhibited nearly identical trends across all code groups.

**Effect of Pretreatments:** The G established baseline values of 8.71 N/mm^2^ for MOR and 6008 N/mm^2^ for MOE. The G1 group resulted in no statistically significant deviation (*p* > 0.05 for both metrics), indicating that the removal of mild surface impurities with hot water was insufficient to alter the bulk mechanical stiffness or strength.

Conversely, the G2 group displayed a consistent decline, with MOR dropping to 8.10 N/mm^2^ and MOE to 5850 N/mm^2^. This mechanical loss directly corroborates the physical degradation observed in the previous sections. The “double alkali effect,” which was identified as the cause of density loss and increased porosity, evidently compromised the intrinsic structural integrity of the fibers as well. It appears that the aggressive chemical attack went beyond surface modification, damaging the cellulose backbone and reducing the fiber’s tensile capacity. This phenomenon aligns with the degradation mechanism described by Mohr et al. (2005) [27], where high alkalinity is shown to decompose the hemicellulose and lignin, leading to fiber embrittlement.

**Effect of Fiber Reinforcement:** The introduction of fibers created a clear bifurcation in mechanical performance, strictly dependent on the fiber type.

Glass fiber reinforcement (G3–G5) effectively transformed the brittle geopolymer matrix into a more structural composite. First, the stiffness enhancement follows the rule of mixtures [28], as high-modulus glass fibers carry a larger portion of the applied load. In addition, under highly alkaline conditions, partial activation of the glass fiber surface is plausible, which may promote the formation of a denser interfacial region and further enhance load transfer, delaying premature failure.

In stark contrast, the inclusion of Hemp Fiber (G6–G8) induced a precipitous drop in mechanical capacity, with the G8 group losing nearly 42% of its stiffness compared to the control. This degradation extends beyond the naturally lower stiffness of organic fibers; it is a structural consequence of the “porosity-strength trade-off.” The agglomeration issues identified in the physical analysis here manifest as mechanical flaws: the fiber clusters act as stress concentrators rather than reinforcements [29]. Consequently, the material behaves less like a dense solid and more like a cellular solid. While this porous architecture severely limits load-bearing capacity, it is the exact feature responsible for the superior thermal insulation (0.10 W/m·K) reported earlier. Thus, the mechanical loss in HF groups should be interpreted not merely as a failure, but as the functional cost of achieving high thermal resistance.

##### Internal Bond (IB) Strength

**Figure 7:**
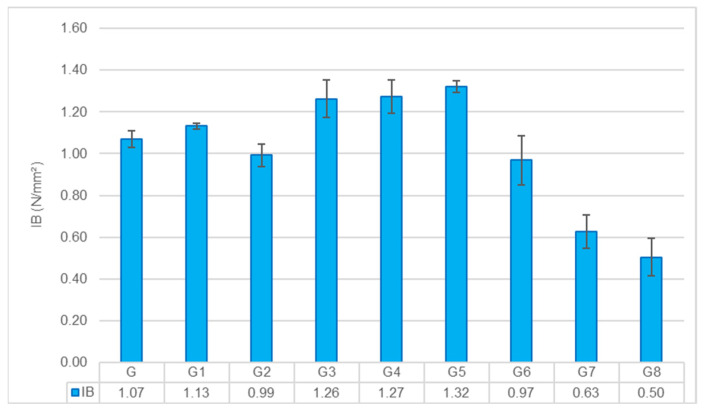
Internal Bond (IB) strength results across all composite groups.

The IB strength acts as a litmus test for the compatibility between the reinforcement and the matrix, effectively measuring how well the composite holds together under perpendicular tension. The results in Figure 7 reveal a stark divergence in performance, dictated largely by interfacial chemistry and physical packing.

**Effect of Pretreatments:** The Control group set a baseline of 1.07 N/mm^2^. Interestingly, the G1 group provided a modest boost, raising the value to 1.13 N/mm^2^. This suggests that simply washing away surface waxes and pectins improved the fiber’s “wettability,” allowing the geopolymer paste to penetrate surface micro-roughness and mechanically interlock more effectively [30].

Conversely, the G2 group displayed a decline to 0.99 N/mm^2^. This drop mirrors the trends seen in the flexural tests and reinforces the “double alkali degradation” hypothesis. The aggressive alkali attack likely rendered the fiber surface friable, creating a “weak boundary layer” rather than a strong interface. Instead of transferring stress, this damaged outer layer likely peeled off under tension, leading to premature failure. This phenomenon is consistent with observations in cementitious composites, where excessive alkalization degrades the fiber cell wall, weakening the fiber-matrix transition zone [31].

**Effect of Fiber Reinforcement:** The fiber type created a clear bifurcation in internal cohesion. Glass Fiber reinforcement (G3–G5) demonstrated superior performance, peaking at 1.32 N/mm^2^ in the G5 group. This robust bonding is not coincidental; it stems from the natural chemical affinity between the silica-rich glass surface and the aluminosilicate matrix. The high alkalinity of the geopolymer likely activated the glass surface, fostering the growth of hybrid C-A-S-H gels that fused the phases together [32]. Furthermore, the CaCl_2_ surface spray likely played a synergistic role; by accelerating the setting reaction at the interface, it effectively densified the contact zone, further locking the structure together [14].

In stark contrast, the Hemp Fiber groups (G6–G8) faced significant structural challenges. As the fiber loading increased, the internal bond strength progressively deteriorated, reaching a minimum of 0.50 N/mm^2^ in the G8 group. This drastic drop offers a clear microstructural explanation for the material’s lower mechanical capacity. The issue is primarily physical: at high volumes, hemp fibers tend to agglomerate, creating “dry clusters” that the viscous geopolymer binder cannot penetrate. These clusters act as internal flaws or voids. Without a continuous matrix to bridge these gaps, the material lacks internal unity and fails easily under tension. This difficulty in wetting and infiltrating dense natural fiber bundles is a well-documented challenge in geopolymer composites [33,34].

### 3.3. Microstructural Characterization: FTIR Analysis

**Figure 8:**
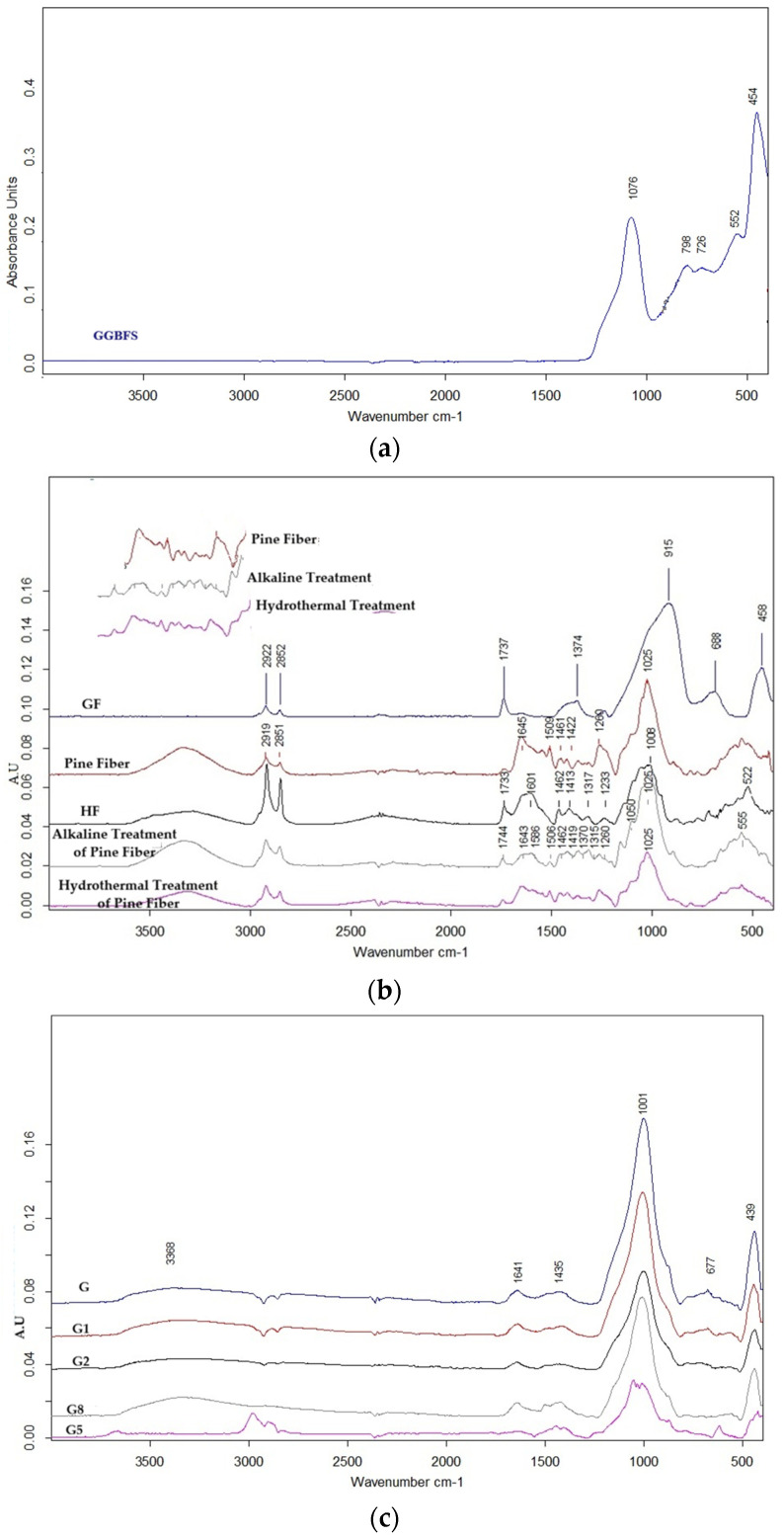
FTIR spectra tracking the chemical evolution of the composites: (**a**) Raw GGBFS precursor, (**b**) Comparative spectra of fibers showing the effect of pretreatments (disappearance of 1737 cm^−1^ peak in NaOH) and fiber type, (**c**) Final geopolymer composites illustrating the gel formation shift (~908 to ~996 cm^−1^).

FTIR was employed to provide molecular evidence underpinning the macroscopic performance observed in the physical and mechanical tests. The spectral evolution from raw precursor to final composite is detailed in Figure 8.

**Precursor Baseline:** The GGBFS precursor (Figure 8a) established the chemical baseline with a dominant band centered at 908 cm^−1^, characteristic of the asymmetric stretching vibrations of Si–O–T (where T = Si or Al) bonds in its amorphous structure. This band serves as the primary reference point; its subsequent shift is the key indicator of the degree of geopolymerization.

**Effect of Pretreatments (Figure 8b):** The impact of fiber pretreatment is clearly visible in the spectral fingerprints of the fibers. The raw hemp fiber exhibits a typical cellulosic profile, distinguished by a sharp ester carbonyl (C=O) peak at 1737 cm^−1^, derived from hemicellulose and lignin components. The evolution of this peak offers a molecular rationale for the mechanical divergence observed between the pretreated groups.

In the NaOH-treated fibers, this peak vanishes completely. While this confirms the successful removal of non-cellulosic binders [35], it simultaneously supports the “double alkali degradation” hypothesis proposed earlier. The aggressive stripping of these binders likely left the cellulose backbone exposed and vulnerable to further attack by the alkaline matrix, rendering the fiber surface friable and leading to the poor interfacial bonding seen in the G2 group. Conversely, the Hot Water treatment resulted in only a marginal intensity reduction in this peak, indicating a milder cleaning process. This preserved the structural integrity of the fiber while sufficiently cleaning the surface to enhance wettability, aligning with the improved performance of the G1 group.

**Geopolymer Network Formation (Figure 8c):** The transformation from loose precursor to hardened composite is evidenced by a definitive spectral shift. Across all groups, the main GGBFS band shifts from 908 cm^−1^ to approximately 990–1000 cm^−1^. This shift toward higher wavenumbers confirms the dissolution of the precursor and its reprecipitation into a more polymerized and connected aluminosilicate gel network (C-A-S-H type) [31].

However, the quality of this gel network varied significantly depending on the reinforcement. The Glass Fiber groups (e.g., G5) displayed a clean, sharp main band, suggesting that the silica-rich glass fibers integrated seamlessly with matrix chemistry, facilitating a continuous and dense network.

In contrast, the Hemp Fiber groups (e.g., G8) exhibited broadening and intensity variations in this region. This disruption suggests a “chemical competition” mechanism. The hydrophilic nature of hemp fibers likely absorbed a portion of the activator water, locally altering the liquid-to-solid ratio and hindering the geopolymerization reaction around the fiber. Furthermore, dissolved organic compounds (such as sugars or extractives) from the natural fibers may have interfered with the nucleation of the C-A-S-H gel, a phenomenon known to retard setting and weaken the matrix structure [34]. This molecular-level disruption explains the voids and reduced mechanical strength observed in the high-fiber composites.

### 3.4. Mineralogical Characterization: XRD Analysis

**Figure 9:**
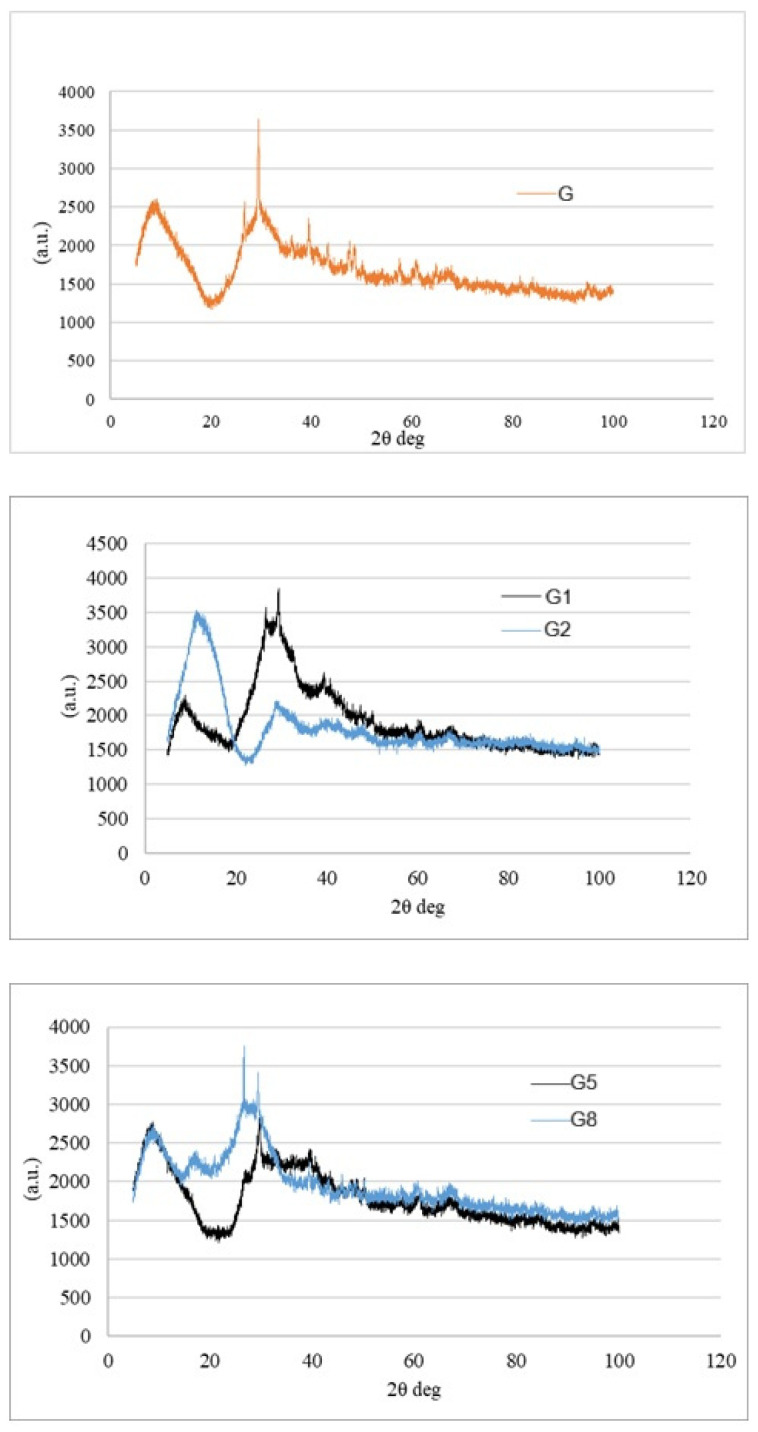
XRD patterns of G, G1, G2, G5, and G8 specimens (intensity in a.u., 2θ in degrees).

X-Ray Diffraction (XRD) was employed to map the crystallographic architecture of the composites, serving as a structural cross-check for the chemical (FTIR) and mechanical findings. The diffraction patterns for all groups are presented in Figure 9.

The unifying feature across all diffractograms is a broad, diffuse hump located between 25° and 35° (2θ). This “amorphous halo” is the signature of a successful geopolymerization [36], confirming that the GGBFS precursor has largely dissolved and re-precipitated into a disordered N-A-S-H or C-A-S-H gel network [6,7]. The visible peaks are attributed to the residual crystalline phases of the unreacted slag (e.g., quartz, calcite) and the semi-crystalline nature of the cellulose fibers, rather than new crystalline geopolymer products.

**Effect of Pretreatments:** The G and G1 groups exhibit nearly identical profiles, dominated by the amorphous geopolymer hump with minor sharp peaks attributed to residual crystalline phases (e.g., calcite or quartz) from the raw slag. The lack of new peaks in G1 confirms that the hot water treatment was a surface-level modification; it cleaned the fibers without altering the fundamental phase composition of the composite.

However, the G2 presents a distinct anomaly. Superimposed on the amorphous hump are sharp, prominent peaks at approximately 15–17° and 22–23° (2θ). These correspond to the crystalline planes of Cellulose I [37]. Their emergence here is structurally significant. It indicates that the aggressive alkali treatment stripped away the amorphous outer layers (hemicellulose and lignin), thereby increasing the “crystallinity index” of the remaining fiber. While this suggests a purer cellulose skeleton, the sharpness of these peaks also hints at a lack of chemical integration; the fibers remain as distinct crystalline inclusions rather than participating in the geopolymer gel formation [38].

**Effect of Fiber Reinforcement:** The impact of fiber type created a sharp crystallographic divergence, mirroring the mechanical results. The G5 maintained a predominantly amorphous profile, indistinguishable from the control matrix. Since glass fibers are inherently amorphous and silica-rich, they blended seamlessly into the geopolymer gel. This lack of distinct crystalline peaks is a positive indicator of homogeneity; it implies that the reinforcement and the matrix have formed a unified, continuous solid, which explains the superior load-bearing capacity (MOR/MOE) of this group [39].

In contrast, the Hemp Fiber group (G8) mirrors the crystallographic signature of the G2 pattern, displaying the characteristic Cellulose I peaks. Here, the signal intensity is directly a function of volume and physical packing. As widely reported in natural fiber geopolymer systems, high fiber loading increases the probability of agglomeration, creating fiber bundles that the viscous binder cannot fully penetrate [21].

These zones of “concentrated crystallinity” effectively act as interruptions in the amorphous binder. Wherever a sharp cellulose peak appears in the diffractogram, it signifies a local break in the continuous C-A-S-H gel network. Instead of a unified load-bearing matrix, the structure becomes a composite of rigid inclusions within a disrupted gel, a phenomenon known to introduce critical defects and reduce mechanical cohesion [40]. Thus, the XRD data provides the final structural evidence for the “porosity-strength trade-off”: the very features that disrupt the dense geopolymer network—crystalline cellulose clumps—are the root cause of the material’s mechanical decline.

### 3.5. Morphological Analysis: SEM

**Figure 10:**
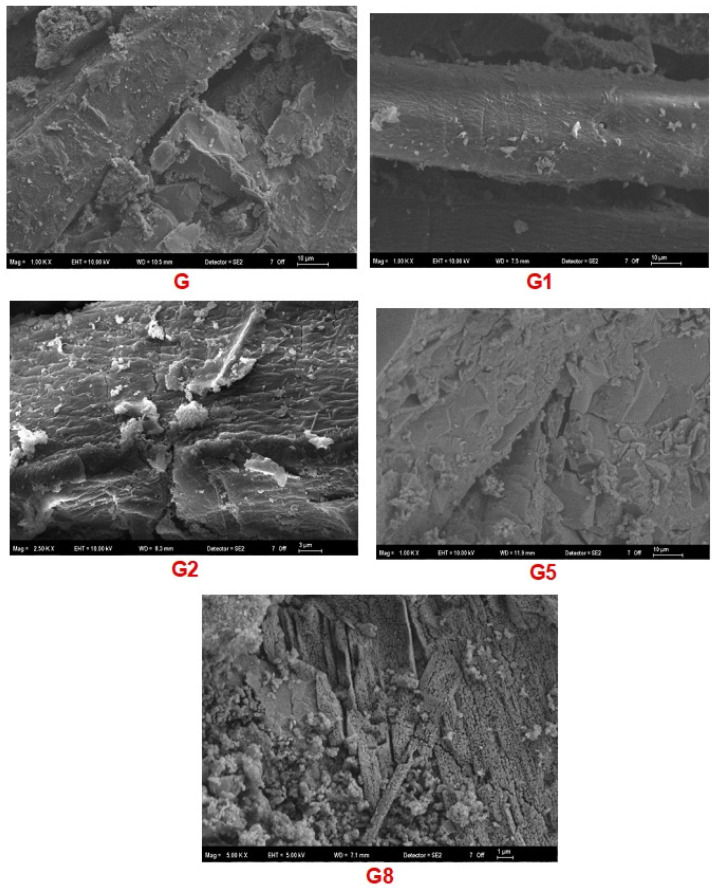
SEM micrographs acquired in SE2 mode showing the interfacial morphology of: (G) Control, (G1) Hot-water pretreatment, (G2) Alkali pretreatment, (G5) Glass fiber reinforcement, and (G8) Hemp fiber reinforcement.

SEM was conducted to provide the visual verification of the fiber–matrix interface and the internal microstructure. These micrographs, presented in Figure 10, serve as the morphological proof connecting the chemical evolution (FTIR/XRD) to the macroscopic performance (Mechanical/Physical).

**Effect of Pretreatments:** The G micrographs reveal a baseline microstructure where the matrix appears relatively compact, yet the fiber–matrix interface is not fully optimized. Small voids and partial detachments suggest that without treatment, the natural waxes on the fiber surface hinder complete wetting by the geopolymer paste.

A subtle but critical improvement is observed in the G1 group. The fiber surfaces appear cleaner and the interface tighter compared to the control. This visual evidence supports the “enhanced wettability” hypothesis discussed earlier; by washing away water-soluble impurities like pectin, the treatment allowed the matrix to mechanically interlock more effectively with the fiber [35]. This cleaner surface morphology explains the slight boost in IB strength observed in the mechanical tests.

In stark contrast, the G2 group exhibits signs of severe microstructural distress. The images reveal fibers with eroded surfaces, fibrillation, and significant detachment from the matrix. Large voids and cracks at the interface act as visual confirmation of the ‘double alkali degradation’ [23]. The aggressive chemical attack stripped the fiber’s protective layers, leaving it structurally compromised and prone to fibrillation [41]. These wide interfacial gaps are the direct physical cause of the low density and poor load-bearing capacity recorded for this group.

**Effect of Fiber Reinforcement:** A significant change in morphology was observed depending on the reinforcement used. Specifically, G5 samples displayed a dense microstructure with a continuous ITZ, where the matrix completely encapsulated the glass fibers without voids. This observation confirms the chemical compatibility suggested by FTIR results and agrees with Korniejenko et al. (2016) [42], who noted that silica-rich fibers foster a more compact geopolymer structure. The lack of voids explains why this group achieved the highest mechanical strength and water resistance; the composite behaves as a unified, monolithic solid.

Conversely, the G8 microstructure is dominated by heterogeneity. The images clearly show fiber agglomeration—clusters of fibers bundled together—surrounded by macro-voids. These ‘dry clusters’ prevented the viscous geopolymer paste from penetrating between individual fibers, leaving large air pockets within the composite [21]. This morphology is the visual definition of the ‘porosity-strength trade-off’ mentioned throughout the study. The voids seen here are responsible for the high water absorption and the material’s failure to transfer stress effectively, resulting in the low MOR and MOE values [43].

## 4. Conclusions

This study explored the potential of valorizing industrial GGBFS waste to produce sustainable geopolymer composites, revealing that the performance of these materials is heavily dependent on the delicate balance between matrix chemistry and reinforcement type. The following specific conclusions are drawn:

1. Pretreatment Threshold: Contrary to conventional polymer composites, aggressive alkaline pretreatment (1% NaOH) proved detrimental in the high-pH geopolymer matrix. The cumulative alkali attack degraded the pine fiber structure, reducing MOR by approximately 7% compared to the control. Mild hot water treatment is recommended as it cleans surface impurities without compromising fiber integrity.
2. Reinforcement Trade-off: A clear performance dichotomy was observed. Glass fibers (9%) maximized structural integrity (MOR: 10.05 N/mm^2^) due to their chemical compatibility. In contrast, Hemp fibers (9%) functioned as effective insulating fillers (λ: 0.10 W/m·K) but significantly reduced mechanical strength due to agglomeration.
3. Future Directions: The results suggest that while 100% GGBFS is a viable matrix, the alkalinity of the activator solution must be carefully balanced. Future research should focus on optimizing the activator concentration to mitigate fiber degradation and exploring hydrophobic coatings for natural fibers to resolve the water absorption issues observed in hemp-reinforced series.

## Figures and Tables

**Table 1:** Experimental design and coding of the produced fiberboards.

Board Code	Fiber Pretreatment	Additive Fiber	Additive Ratio (%)	Fiber/Binder Ratio
**G (Control)**	None (Raw Pine)	–	–	1/9
**G1**	Hot Water	–	–	1/9
**G2**	1% NaOH	–	–	1/9
**G3**	None	Glass Fiber	3	1/9
**G4**	None	Glass Fiber	6	1/9
**G5**	None	Glass Fiber	9	1/9
**G6**	None	Hemp Fiber	3	1/9
**G7**	None	Hemp Fiber	6	1/9
**G8**	None	Hemp Fiber	9	1/9

**Table 2:** Chemical composition of the GGBFS binder (wt%).

Component	CaO	SiO_2_	Al_2_O_3_	MgO	SO_3_	Fe_2_O_3_	Others
GGBFS	38.5	36.5	12.9	4.45	1.89	0.86	4.90

**Table 3:** Chemical composition of the natural fibers used in the study.

Fiber Type	Cellulose (%)	Hemicellulose (%)	Lignin (%)	Ash/Extractives (%)
Pine	45.2	25.4	27.9	1.5
Hemp	67.5	21.8	4.2	6.5

**Table 4:** Physical properties of the GGBFS-based wood composites (Mean ± SD).

Group Name	Code	d (g/cm^3^)	TS (%)	WA (%)	Thermal Conductivity (W/m·K)
Control	G	1.44 ± 0.02	3.0 ± 0.2	9.0 ± 1.2	0.15
Hot Water	G1	1.43 ± 0.01	2.0 ± 0.1	8.0 ± 0.5	0.16
NaOH Treated	G2	1.41 ± 0.01	3.0 ± 0.3	10.0 ± 0.8	0.18
3% Glass Fiber	G3	1.45 ± 0.01	2.0 ± 0.1	8.0 ± 1.0	0.15
6% Glass Fiber	G4	1.47 ± 0.01	2.0 ± 0.1	8.0 ± 0.6	0.16
9% Glass Fiber	G5	1.49 ± 0.01	1.0 ± 0.1	7.0 ± 0.4	0.15
3% Hemp Fiber	G6	1.38 ± 0.01	3.0 ± 0.4	11.0 ± 1.1	0.14
6% Hemp Fiber	G7	1.36 ± 0.04	3.0 ± 0.5	12.0 ± 1.3	0.12
9% Hemp Fiber	G8	1.34 ± 0.02	3.0 ± 0.8	13.0 ± 1.4	0.10

**Table 5:** Mechanical properties of the geopolymer composites (Mean ± Standard Deviation).

Code	MOE (N/mm^2^)	MOR (N/mm^2^)	IB (N/mm^2^)
G	6008.40 ± 192.37	8.71 ± 0.25	1.07 ± 0.04
G1	6002.80 ± 71.36	8.86 ± 0.53	1.13 ± 0.01
G2	5850.40 ± 138.74	8.10 ± 0.06	0.99 ± 0.05
G3	6149.80 ± 170.23	9.20 ± 0.18	1.26 ± 0.09
G4	6300.00 ± 171.39	9.80 ± 0.08	1.27 ± 0.08
G5	6499.80 ± 304.97	10.05 ± 0.23	1.32 ± 0.03
G6	5132.00 ± 170.16	7.41 ± 0.28	0.97 ± 0.12
G7	4846.80 ± 74.19	7.16 ± 0.74	0.63 ± 0.08
G8	3489.40 ± 297.67	5.77 ± 0.53	0.50 ± 0.09

## Data Availability

The original contributions presented in the study are included in the article; further inquiries can be directed to the corresponding author.

## Abbreviations

The following abbreviations are used in this manuscript:

GGBFS	Ground Granulated Blast Furnace Slag
GF	Glass Fiber
HF	Hemp Fiber
NaOH	Sodium Hydroxide
Ms	Silica Modulus
D	Density
WA	Water Absorption
TS	Thickness Swelling
MOR	Modulus of Rupture
MOE	Modulus of Elasticity
IB	Internal Bond
SEM	Scanning Electron Microscopy
XRD	X-ray Diffraction
XRF	X-ray Fluorescence
FTIR	Fourier Transform Infrared Spectroscopy
